# Unusual Presentation of Hydatid Cyst in Breast with Magnetic Resonance Imaging Findings

**DOI:** 10.1155/2017/6237435

**Published:** 2017-01-11

**Authors:** Ali Koc, Inanc Samil Sarici, Umit Erkan Vurdem, Ozgur Karabiyik, Ummugulsum Ozgul Gumus

**Affiliations:** ^1^Department of Radiology, Kayseri Training and Research Hospital, Kayseri, Turkey; ^2^Department of General Surgery, Kanuni Sultan Suleyman Training and Research Hospital, İstanbul, Turkey

## Abstract

We report a case of 59-year-old woman with a painful left breast mass, compatible with types II-III hydatid cyst. Lesion was evaluated with mammography, ultrasound, computed tomography, and magnetic resonance imaging modalities. Magnetic resonance imaging had important diagnostic role with demonstrating characteristic features of the lesion and had capability of showing complications. Surgery also confirmed the diagnosis of a hydatid cyst.

## 1. Introduction

Hydatid cyst of the breast is very rare. Patients usually present to the hospital with a palpable and painless lump in the breast. It is challenging to differentiate it from other tumoral lesions of the breast. Only few reports of breast hydatid cyst are published and majority of the reported cases have been diagnosed postoperatively. We report a case with breast hydatid cyst with MR imaging findings. The report has been approved by the Institutional Review Board of the institution with patient informed consent.

## 2. Case Report

A 59-year-old female patient referred with palpable, longstanding (over 10 years) mass in the periphery of left breast that was remarkable with pain for the last 8 months. There was no history of breast trauma, hormone replacement therapy, or family history of malignancy. Physical examination revealed a palpable nonfixed mass on the anterior chest wall, peripherally located in the left breast with regular borders. The nipple, areola, and overlying skin were normal and no palpable lymph node in both axilla. Routine laboratory tests were in normal ranges.

**Figure 1:**
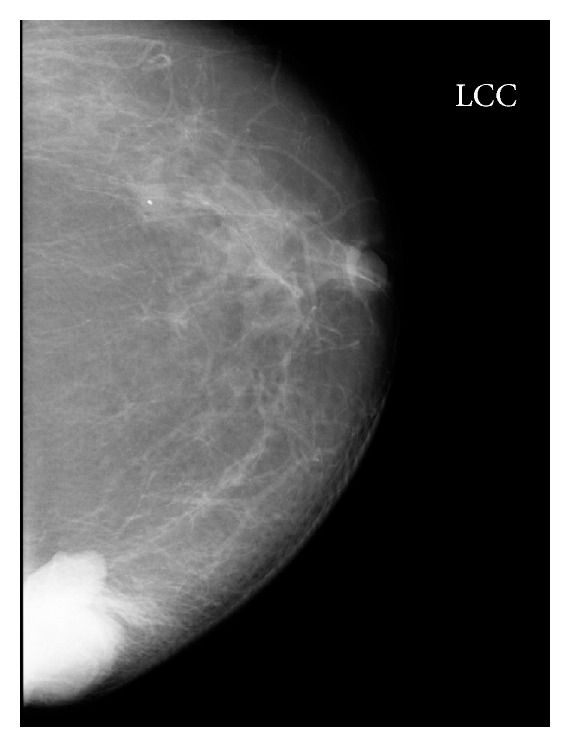
59-year-old woman with left breast mass. CC mammogram shows dense mass with sharply defined, lobulated contours, located at the inner part of the left breast.

Mammography examination with craniocaudal (CC) and medial lateral oblique (MLO) projections revealed dense mass, situated peripherally at 9 o'clock, with a regular, lobulated contour in the left breast, with no identifiable micro- and macrocalcifications (Figure 1).

**Figure 2:**
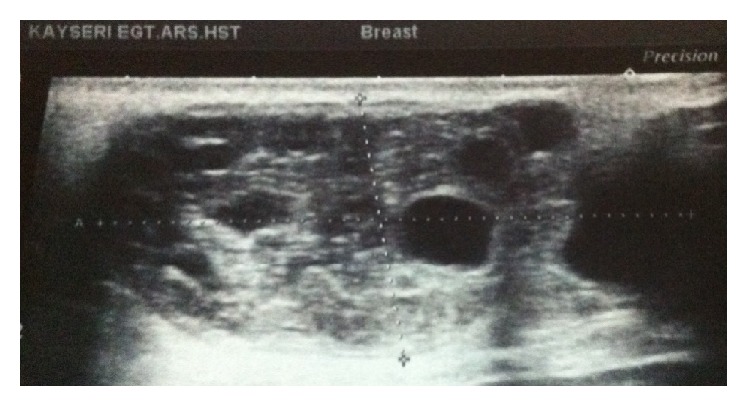
Gray-scale sonogram. Smooth bordered semisolid mass with internally scattered, mostly circumferentially oriented anechoic multiple cyst, creating rosette appearance and accompanying echogenic material filled the intercystic spaces (hydatid sand).

At sonography, lesion was compatible with a semisolid mass and had a smooth bordered, moderately thickened wall, with a dimension of 4.5 × 3.3 × 2.9 cm, containing internal detached membrane, giving an appearance of “water lily.” Lesions have contained circumferentially oriented anechoic millimetric loculations divided with septations, resembling daughter cysts. Intercystic spaces were filled with homogenous echogenic material (hydatid sand) predominantly seen in the centre of the lesion, creating rosette appearance (wheel-spoke pattern) (Figure 2). Doppler sonography showed no internal vascularity. No significant enlarged axillary lymph nodes were detected with both examination.

**Figure 3:**
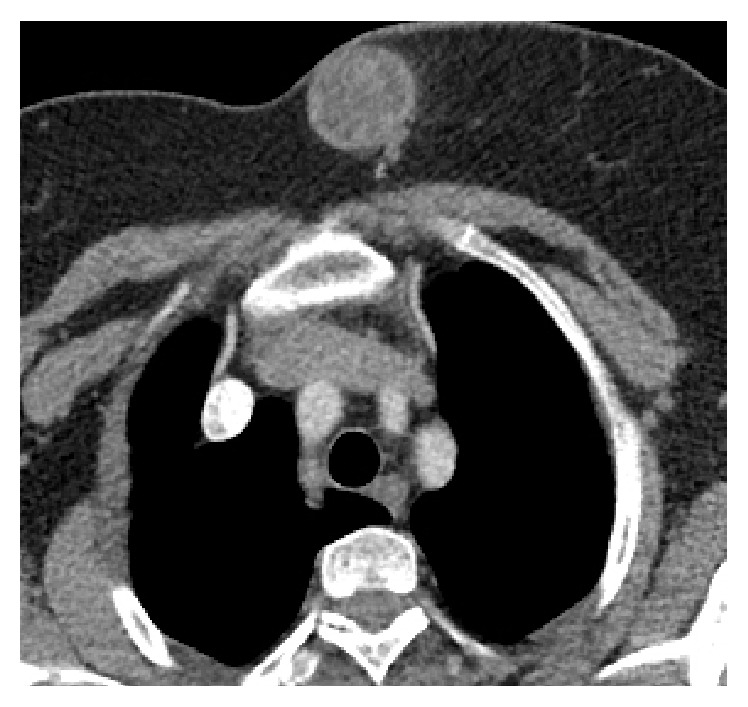
Axial contrast-enhanced thorax CT image. Hypodense, well-defined mass with capsular wall and internal septae, situated at the inner part of the left breast. The lesion shows capsular enhancement.

**Figure 4:**
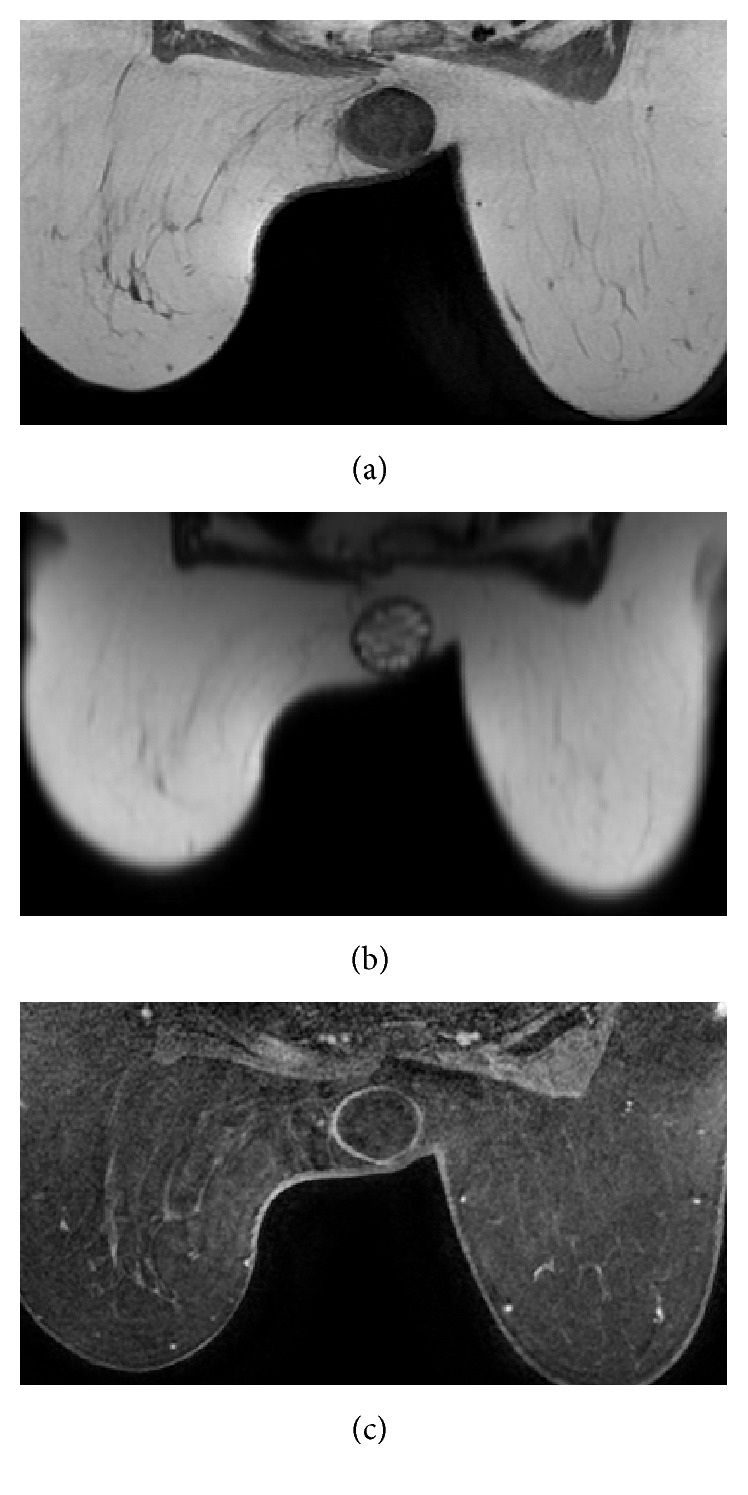
(a) Axial T1- and (b) T2-weighted MR images show that left breast mass with well-defined borders has multiple cystic loculations creating “rosette” pattern. Capsular wall and internal septae of the lesion are isointense on T1-weighted image and hypointense on T2-weighted image; marginally contrast enhancement seen on fat-saturated contrast enhanced T1-weighted image (c).

**Figure 5:**
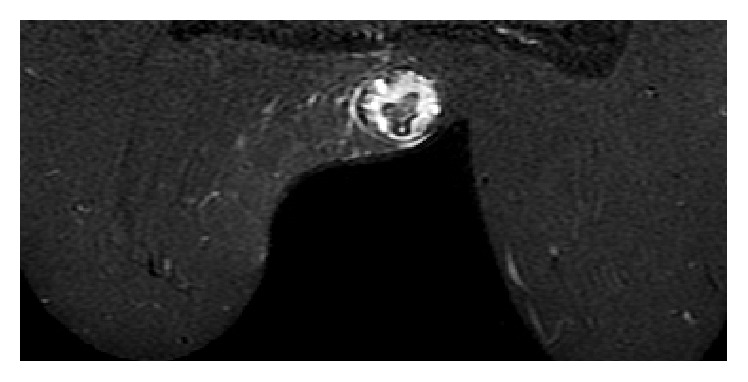
STIR sequence image demonstrates hypointense capsule and detached internal membrane with perilesional fluid accumulation eccentrically, revealing focal rupture.

**Figure 6:**
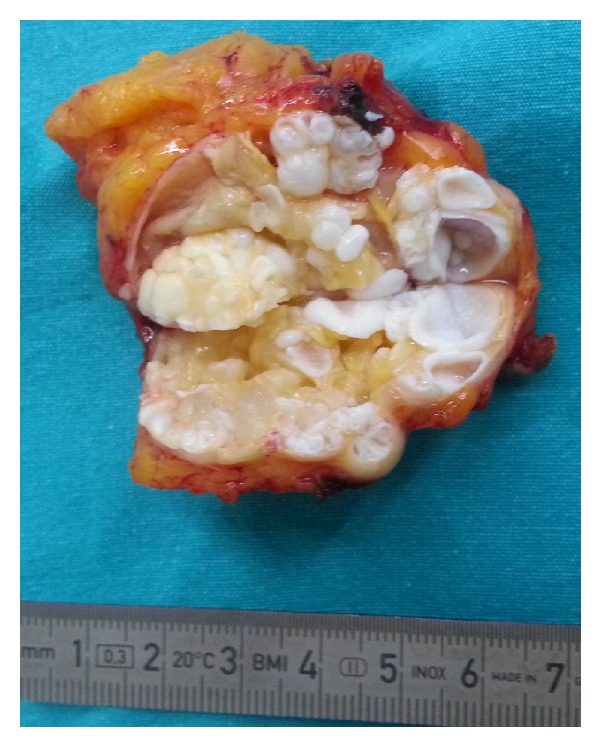
The surgical specimen showed characteristic daughter cysts of hydatid disease.

Because the sonographic findings suggested hydatid cyst, thoracoabdominal CT was performed for checking probable simultaneous lesions. CT exam showed no abnormal mass in performed body parts, without left breast lesion. The breast lesion was heterogeneous hypodense with a smooth thick wall and septations, internally, had a density of 35 HU (Figure 3). Dynamic contrast enhanced MRI (1.5 Tesla) with contrast enhancement pattern was performed for further evaluation and detailed structural analysis of mass. At MRI, capsular wall of the mass was smooth and moderately thickened. There were internally scattered, mostly circumferential oriented small loculations, which separated with thin septations, giving an appearance of rosette or wheel-spoke. These nonenhancing multiloculations had low signal intensity on T1-weighted and high signal intensity on T2-weighted images compatible with daughter cysts. Intercystic spaces were filled with material, which was isointense with muscle tissue on T1-weighted and hyperintense on T2-weighted images, suggesting hydatid sand. Infolded membrane at the inferior pole of lesion gave an appearance of “water lily” sign. The capsular wall and infolded membrane were isointense on T1-weighted and hypointense on T2-weighted images where hypointensity was remarkable with* Short Tau Inversion Recovery* (STIR) images. The capsular wall of the lesion enhanced marginally as smooth rim with a gradually increasing pattern, whereas internal septations and detached membrane were not enhanced at dynamic T1-weighted images (Figures 4(a), 4(b), and 4(c)). Superior and inferior polar outpouchings of the mass and capsular defect at the inferior edge were noteworthy. Mass was outlined with a curvilinear, minimal fluid collection near the defect, revealing a focal rupture (Figure 5). The surgical specimen showed characteristic daughter cysts of hydatid disease (Figure 6).

## 3. Discussion

*Echinococcus granulosus* is the most common cause of hydatid disease in humans. The location is mostly in the liver (75%) and lungs (15%), with only 10% occurring in other parts of the body; in the breast it only accounts for 0.27% of all cases. Hydatid disease of the breast is extremely rare even in endemic areas; it can be the only primary site or part of disseminated hydatidosis. Patients usually present with a palpable and painless lump in the breast. Differentiation of lesion from other tumors can be challenging. Only few reports are published and majority of the reported cases have been diagnosed postoperatively [1–5]. In our case, breast lesion was painful for the last 8 months that had a history of 10 years which gradually enlarged.

The imaging modalities for diagnosis of breast hydatid disease are mammography, ultrasound, and MRI. Mammography shows a nonspecific, homogeneous, smooth, circumscribed lesion [6]. When secondary infection occurs, hydatid cyst cannot be distinguished from breast abscess, clinically by mammography [7]. In our case, mammography showed nonspecific dense mass with a smooth, lobulated contour without micro- and macrocalcification. The lobulation of contour was remarkable with eccentric outpouchings.

The ultrasound findings vary according to the degree of maturation and the complications [7]. Gharbi et al. [8] have described five types of ultrasound findings for hydatid cysts. Types II and III hydatid cysts have more reliable diagnostic imaging properties than other types. Multiple daughter cysts separated by a fluid matrix that contains a mixture of membranes of broken daughter vesicles, scolices, and hydatid sand with mixed echogenicity may give rise to a “wheel-spoke” pattern. Separation of the ruptured endocyst layer from the ectocyst leads to a free floating membrane which produces the so-called water lily sign [7, 9].

In our case, sonography revealed well-demarcated mass with lobulated contours that contain round-shaped cysts scattered circumferentially and septae within, giving an appearance of “water lily” sign and “wheel-spoke” pattern of the disease, consistent with a types II-III hydatid cyst.

MRI findings can be helpful but not specific. The findings of cystic lesion with capsular enhancement are suggestive of hydatid cyst. The capsular enhancement is more typical with secondary infection. Diagnosis is frequently delayed because no specific signs are found at the time of examination, and they instead mimic other pathologies [5]. MR imaging features are similar to those of a simple cyst and include hypointensity on T1-weighted and marked hyperintensity on T2-weighted images. A low-signal-intensity rim (“rim sign”) is more evident on T2-weighted MR images. At MR imaging, daughter cysts may appear to be hypointense or isointense relative to the maternal matrix on T1- and T2-weighted images. The “serpent sign” or “snake sign,” which represents collapsed parasitic membranes secondary to damage or degeneration, is another MR imaging manifestation of a hydatid cyst. These membranes have a low signal intensity with all sequences [10]. In our case, the cystic lesion represented “water lily” sign and rosette pattern with related fibrous elements (internal septae, detached membrane, and capsule), which were isointense and hypointense on T1- and T2-weighted images, respectively. Hypointensity was more remarkable on STIR images. Only the capsular wall of the cyst enhanced as gradually increasing pattern on contrast-enhanced dynamic T1-weighted images.

Edema or acute inflammation caused by compression of or allergic reaction in soft tissue adjacent to the cyst is uncommon but may be seen [11]. Curvilinear minimal fluid accumulation in the inferior margin of the lesion and focal capsular rupture were only seen at STIR sequence T2-weighted images. MRI allowed to show detailed structure and rupture as a complication, better than other imaging modalities in this case.

The differential diagnosis based on mammography would include cyst, fibroadenoma, phyllodes tumour and, rarely circumscribed carcinoma. Vega et al. noted that the presence of ring shaped structures and interseptal bands in the slowly growing breast mass should suggest a hydatid cyst [6]. While morphology is important and criterion for staging of hydatid disease, imaging modalities having high contrast resolution should be considered in selected cases for investigation. According to stage of the hydatid disease, MRI also had capability to show lesion characteristics and perilesional soft tissue changes better than sonography.

*In conclusion*, hydatid disease of the breast should be considered as a differential diagnosis of breast lumps. Sonography and MRI have an important diagnostic role with giving additional information about internal structure of hydatid cyst rather than CT and mammography. STIR sequence T2-weighted MRI, especially, helps to identify complications (e.g., rupture and secondary infection) in cystic masses.
